# Platelet rich fibrin in treatment of penile skin defects. A case series and review of the literature

**DOI:** 10.1016/j.eucr.2025.103198

**Published:** 2025-09-02

**Authors:** Glenn Lamers, Laurence Royakkers, Stijn Schapmans, Katrien De Coster, Natalia Zabegalina, Johan Van Dyck

**Affiliations:** a Department of Urology, Noorderhart Hospital North Limburg, Maesensveld 1, 3900, Pelt, Belgium; b Department of Urology, University Hospitals Leuven, Herestraat 49, 3000, Leuven, Belgium

**Keywords:** Platelet rich fibrin, Reconstructive urology, Regeneration, Urologic surgery

## Abstract

Treatment of complex and chronic wounds often remains a challenge for clinicians, carrying a severe impact on the wellbeing of patients and the health care system. Leukocyte-and Platelet rich fibrin (L-PRF) is an autologous platelet product with regenerative properties that can be used to stimulate and accelerate wound healing. Its usage in oral surgery and dentistry is well established but evidence in urologic surgery is limited. We report two cases with extensive penile cutaneous defects after surgical debridement, treated with L-PRF technology and review the available literature regarding its utilization in the field of urology.

## 1 Introduction

Platelet-rich fibrin (PRF) is a second-generation autologous platelet concentrate. Platelet concentrates in regenerative medicine are classified into pure platelet-rich plasma (P-PRP), leukocyte- and platelet-rich plasma (L-PRP), pure platelet-rich fibrin (P-PRF), and leukocyte- and platelet-rich fibrin (L-PRF) based on fibrin architecture and leukocyte content. These are developed to increase wound healing by encouraging epithelial growth.^1^ Although a relatively new technology, its use and benefit have been extensively researched and established in maxillofacial surgery and dentistry.^2^ The potential benefits in wound healing achieved in this discipline have also been studied in several other surgical specialties, although with varying results.^3^ In every surgical discipline, faster and more efficient wound healing is coveted, and chronic wound problems can be a serious burden on the patient and the health care system in terms of costs, need of care, and quality of life.^4^ In the field of urology, the application of PRF has primarily been investigated in hypospadias repair, with the first case reported by Soyer et al., in 2013 to repair a urethrocutaneous fistula after primary hypospadias repair in a 3-year-old boy.^5^ We present two cases with an extensive penile cutaneous defect after debridement, treated with L-PRF technology. To our knowledge, this is the first report of L-PRF use for penile skin wound healing. We additionally reviewed the literature regarding the usage of PRF in urologic surgery.

## 2 Case presentation

In the first case, a 59-year-old male presented after a fall on the genitalia and perineum, developing an immediate scrotal/perineal hematoma, pain, and urinary retention. His past medical history included arterial hypertension, transurethral resection of the prostate, and bilateral orchidectomy with secondary hypogonadism and osteoporosis, for which he received testosterone supplementation.

Six days later, the patient developed chills and sweats, and scrotal pain was poorly controlled with standard analgesic agents. On physical examination, an organized and painful scrotal and penile hematoma was observed. Scrotal ultrasound revealed air pockets, confirmed by a CT scan. The patient was admitted and started on amoxicillin/clavulanic acid, continued throughout hospitalization. Laboratory results showed only mild inflammatory response, with increased leukocyte count and CRP levels of 74 mg/L. A urethrogram showed no urethral rupture.

**Fig. 1:**
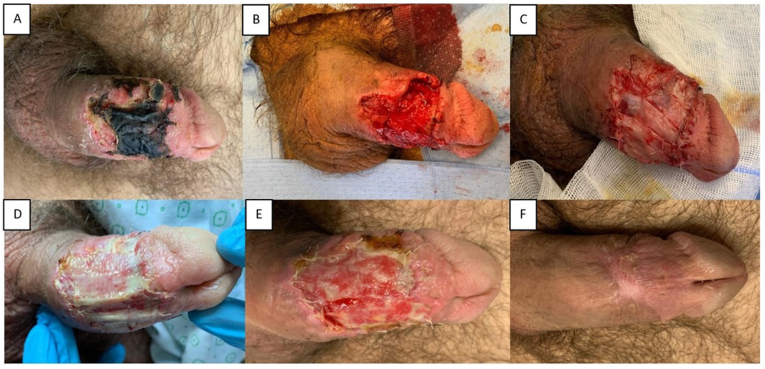
Case 1. Penile necrosis at the ventral and right lateral position (a). Wound after surgical excision and debridement (b). Platelet rich fibrin (PRF) grafts sutured onto the defect (c). Penile wound 4 days (d) and 8 days (e) after first PRF-graft placement. Penile wound 3 weeks after second PRF-graft placement (f).

The following day, surgical drainage was performed. After degloving the penis and removing the hematoma, hemostasis was achieved and a circumcision was performed. Ten days later, the patient developed penile subcoronal necrosis ventrally and right laterally, requiring excision and debridement the next day (Fig. 1a–b). The necrosis extended through the dermis down to Buck's fascia, which was intact and unharmed from necrosis. Considering the large area, the patient was preoperatively counselled on the option of PRF grafting, to which he consented.

To cultivate the L-PRF grafts, blood samples were obtained from a peripheral vein into a 9 mL IntraSpin blood tube (Intra-lock, Birmingham). These samples were immediately placed in an IntraSpin centrifuge (Intra-lock) at 2700 rotations per minute for 12 minutes. The L-PRF clot was then separated from the red blood cells and placed in an Xpression L-PRF box (Intra-lock). By applying slight compression, the clot was transformed into an L-PRF membrane. These were then sutured into the wound with Vicryl 5-0 (Fig. 1, c). Next, a paraffin gauze dressing was applied, chosen because it provides a non-adherent interface and maintains moisture without interfering with growth factor release, and the penile wounds were wrapped in a cohesive elastic bandage.

Over the following week, wound healing progressed well. In several areas, however, granulation tissue was lacking (Fig. 1d–e). Based on the persistent non-granulating areas, the procedure was repeated on day 21. Three weeks after the second PRF graft placement, the wound was completely closed with limited soft central scar tissue (Fig. 1,f). The penile skin surrounding the scar was flexible. Pain control was achieved during the entire follow-up period, and the patient was satisfied with the overall cosmetic results.

**Fig. 2:**
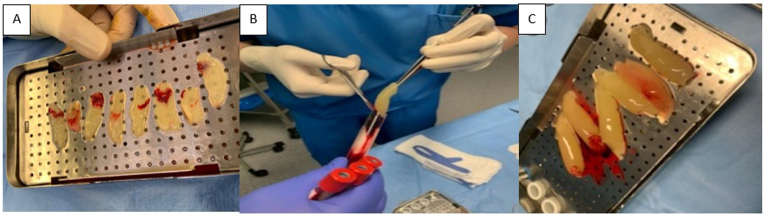
Platelet rich fibrin grafts obtained in case 1 (a). Grafts during (b) and after (c) extraction in case 2.

The obtained L-PRF membranes are shown in Fig. 2.

In the second case, a 30-year-old male presented to the emergency department with penile swelling and ulcers, fever, and general malaise. There was no preceding trauma or unsafe sexual contact. The patient admitted to sporadic use of amphetamine and cannabis, but otherwise his medical history was unremarkable.

**Fig. 3:**
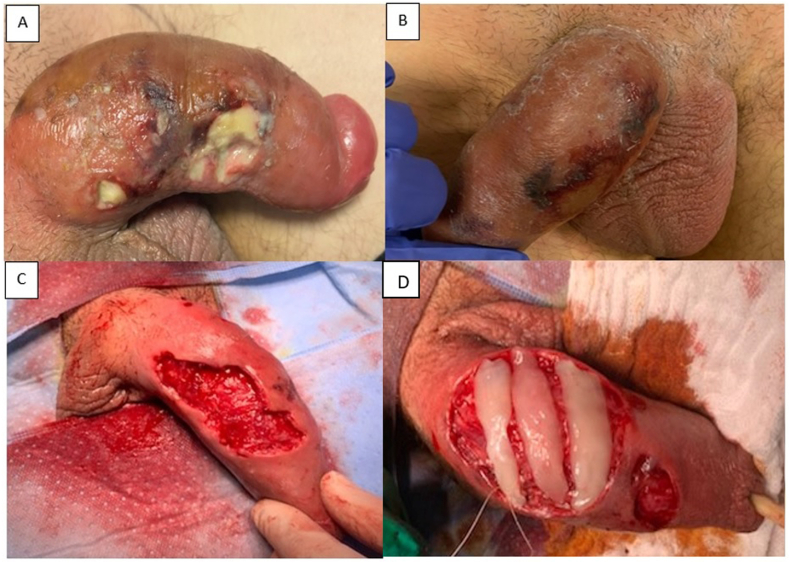
Case 2. Penile ulcerations and cellulitis (a–b). Wound after excision and debridement (c). Platelet rich fibrin grafts sutured onto the defect (d).

On physical examination, several necrotizing wounds were observed at the base of the penis with cellulitis extending to the groin (Fig. 3a and b). A CT scan was performed to rule out Fournier's gangrene. Further diagnostic investigations, including urine and blood cultures, HIV, and sexually transmitted infection (STI) screening, were negative. He was started on IV amoxicillin/clavulanic acid, but after two days, deterioration of the lesions was observed. Therefore, an extensive wound debridement was performed the same day (Fig. 3, c), and the patient consented to PRF grafting (Fig. 3, d).

**Fig. 4:**
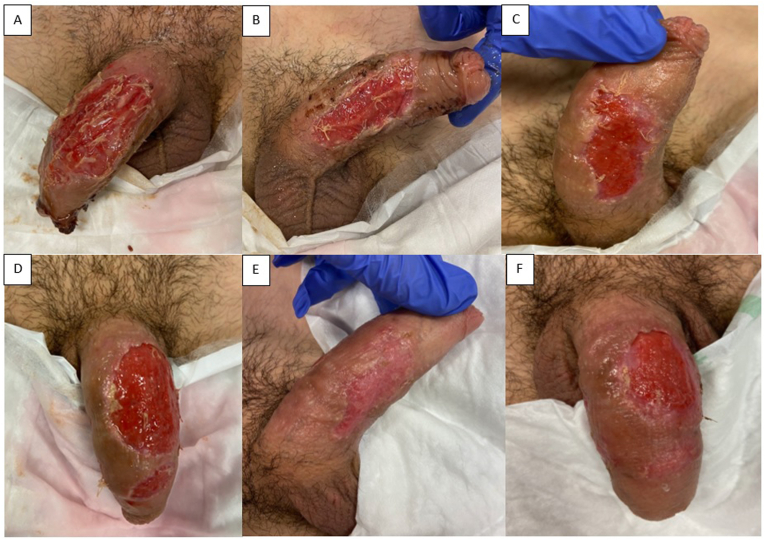
Case 2. Penile wound 6 days (a–b), 13 days (c–d) and 23 days (e–f) after platelet rich fibrin graft placement.

After five days, the dressing was removed (Fig. 4a–b). The patient was discharged with amoxicillin/clavulanic acid for an additional two weeks. In this case, an alginate-based antibacterial moisturizing cream was added to the gauze dressing to reduce bacterial burden and support moist wound healing.

At outpatient wound inspections one week (Fig. 4c–d) and two weeks (Fig. 4e–f) after discharge, healing had progressed well. The defect on one side was nearly closed without fixed scar tissue, and penile skin was remodeling. The contralateral defect remained open but showed healthy granulation tissue. The patient was unfortunately lost to follow-up thereafter.

Written informed consent was obtained from both patients for publication of this case report and accompanying images.

## 3 Discussion

Platelet-rich fibrin is an autologous platelet concentrate obtained from venous blood. It is collected with a venipuncture in a plastic container without an anticoagulant and immediately centrifuged to minimize clotting. Centrifugation for 10–14 minutes at 2700–3000 rpm results in three layers: red blood cells at the bottom, platelet-poor plasma at the top, and a middle fibrin clot containing platelets, leukocytes, and growth factors.^2^This fibrin matrix serves as a scaffold which can be cut to a desired shape and allows sustained release of growth factors, promoting cell migration, angiogenesis, and tissue regeneration.^3^^,^^6^^,^^7^

PRF has been extensively studied in oral and dental surgery, showing improved wound healing and reduced postoperative pain.^2^ In other surgical specialties, results regarding it's increased healing properties vary. For example, PRF has shown little effect in functional outcome after rotator cuff repair,^8^while accelerated healing is seen in tympanic membrane repair, burns, and some chronic wounds.^1^^,^^3^Multiple randomized controlled trials (RCTs) suggest potential benefit in diabetic and venous leg ulcers,^3^^,^^6^particularly with repeated applications, and a recent meta-analysis by Chen et al. confirmed significant clinical benefit in this setting.^9^

We reviewed the available literature regarding PRF in the field of urology. A PubMed search was performed using the search terms “platelet rich fibrin”, “platelet-rich fibrin”, “platelet graft” or “platelet rich plasma” combined with the term “urology”.

Four reports describe the use of autologous platelet concentrate application in hypospadias repair. Soyer et al. first reported successful fistula repair in 2013 with PRF.^5^ Guinot et al. evaluated PRF as a coverage layer in distal hypospadias repair and found no significant difference compared with dartos flap interposition in 33 patients with 2 patients developing a fistula in the follow up period of 18 months.^10^Mahmoud et al., however, studied PRP coverage with dartos flap interposition in a randomized controlled trial of 180 patients. They found no difference in fistula rates (10 vs 13 %, p = 0.64), but did report fewer infections in the PRP group (0 vs 6.7 %, p = 0.03) over a follow-up period of 23.5 months.^11^Eryilmaz et al. performed a RCT in 40 patients and noted lower fistula and stenosis rates in the PRF group (10 vs 25 % and 5 vs 25 %, respectively), but these did not reach statistical significance. Infection rates, however, were significantly reduced.^12^A recent systematic review by Moran et al. included PRF along with other biologic coverings and showed significantly reduced urethrocutaneous fistula rates overall, although PRF-specific conclusions could not be made.^13^The generic principle is similar so it seems a promising alternative.

In addition, there is one study that uses a platelet product related to PRF, platelet rich plasma gel, in buccal graft urethroplasty for bulbar and penile urethral stricture repair. In this report of10 patients there were no complications or re-strictures.^14^

Although L-PRF is not that extensively studies, other platelet concentrates like PRP has been investigated for several urological indications. Most common practices of PRP use in urology are intravesical instillations for interstitial cystitis and intracavernosal injections for erectile dysfunction, both with promising results.^15^^,^^16^ These studies should be clearly distinguished from PRF evidence, although they highlight the regenerative applications of platelet concentrates.

Our two cases represent, to our knowledge, the first reports of L-PRF for penile skin loss. Conventional options for penile skin reconstruction include primary repair, split-thickness or full-thickness skin grafts, local flaps, and conservative measures such as dressings and negative pressure therapy.^17^^,^^18^ The treatment choice depends on wound size, surgical expertise and local resources. L-PRF may offer a solution for modarete wound defects too large for primary closure or where residual penile skin is limited. It can provide advantages of autologous origin, with low cost, and the ability to promote healing without the morbidity of donor-site harvesting. However, its role compared to grafting techniques remains undefined.

To briefly frame the impact of chronic wound care on the health care system in this discussion, Olssen et al. performed a systematics review addressing the issue.^4^ When looking at several developed countries, the mean societal cost per patient per year for chronic wounds is over $10.000. When considering the UK, 3 % of healthcare expenditure is attributed for wound management. Considering these are underestimated numbers, the economic impact of chronic wound care is therefore substantial and every means to reduce costs and treatment time are desired. Platelet rich fibrin is an inexpensive and safe technology that could possibly help to achieve this goal.

Limitations of our report include the short follow-up period, absence of objective endpoints such as validated wound-healing scores, and lack of comparison with standard reconstructive techniques. Future studies with standardized outcomes and controlled comparisons are needed to establish the role of L-PRF in penile wound management.

## 4 Conclusion

Harvesting and applying L-PRF may be a valuable adjunct for wound healing in penile cutaneous defects. This case series demonstrates successful use of L-PRF in two patients with extensive penile skin loss, both achieving satisfactory healing without grafting. While the available urologic evidence in this setting remains limited, PRF has shown promise in other surgical disciplines. It could offer a safe, inexpensive, and autologous alternative to conventional reconstructive approaches in selected cases but comparative studies are needed to establish its comparative efficacy.

## CRediT authorship contribution statement

**Glenn Lamers:** Writing – review & editing, Writing – original draft, Formal analysis, Data curation, Conceptualization. **Laurence Royakkers:** Writing – review & editing, Writing – original draft, Formal analysis, Data curation. **Stijn Schapmans:** Writing – review & editing, Data curation, Conceptualization. **Katrien De Coster:** Writing – review & editing. **Natalia Zabegalina:** Writing – review & editing. **Johan Van Dyck:** Writing – review & editing, Conceptualization.

## Consent for publication

Informed consent was obtained from the patient for the publication of this case report and any accompanying images.

## Ethical considerations

Approval from the ethics committee was not required.

## Financial disclosure

This research did not receive any forms of funding.

## Conflicts of interest

There are no conflicts of interest to be reported by any of the contributing authors.

## References

[1] Nanditha S., Chandrasekaran B., Muthusamy S., Muthu K. (2017). Apprising the diverse facets of Platelet rich fibrin in surgery through a systematic review. Int J Surg.

[2] Fan Y., Perez K., Dym H. (2020). Clinical uses of platelet-rich fibrin in oral and maxillofacial surgery. Dent Clin North Am.

[3] de Carvalho C.K.L., Fernandes B.L., de Souza M.A. (2020). Autologous matrix of platelet-rich fibrin in wound care settings: a systematic review of randomized clinical trials. J Funct Biomater.

[4] Olsson M., Järbrink K., Divakar U. (2019). The humanistic and economic burden of chronic wounds: a systematic review. Wound Repair Regen.

[5] Soyer T., Çakmak M Fau - Aslan M.K., Aslan Mk Fau - Şenyücel M.F., Şenyücel Mf Fau - Kisa Ü., Kisa Ü. (2012). Use of autologous platelet rich fibrin in urethracutaneous fistula repair: preliminary report. Int Wound J.

[6] Miron R.J., Fujioka-Kobayashi M., Bishara M. (2017). Platelet-rich fibrin and soft tissue wound healing: a systematic review. Tissue Eng Part B Rev.

[7] Yu P., Zhai Z., Jin X., Yang X., Qi Z. (2018). Clinical application of platelet-rich fibrin in plastic and reconstructive surgery: a systematic review. Aesthetic Plast Surg.

[8] Mao X.H., Zhan Y.J. (2018). The efficacy and safety of platelet-rich fibrin for rotator cuff tears: a meta-analysis. J Orthop Surg Res.

[9] Chen J., Wan Y., Lin Y., Jiang H. (2022). Platelet-rich fibrin and concentrated growth factors as novel platelet concentrates for chronic hard-to-heal skin ulcers: a systematic review and meta-analysis of randomized controlled trials. J Dermatol Treat.

[10] Guinot A., Arnaud A., Azzis O. (2014). Preliminary experience with the use of an autologous platelet-rich fibrin membrane for urethroplasty coverage in distal hypospadias surgery. J Pediatr Urol.

[11] Mahmoud A.Y., Gouda S., Gamaan I., Baky Fahmy M.A. (2019). Autologous platelet-rich plasma covering urethroplasty versus dartos flap in distal hypospadias repair: a prospective randomized study. Int J Urol.

[12] Eryilmaz R., Şimşek M., Aslan R. (2020). The effect of plasma rich platelet graft on post-operative complications in mid-penile hypospadias. Andrologia.

[13] Moran G.W., Kurtzman J.T., Carpenter C.P. (2022). Biologic adjuvant urethral coverings for single-stage primary hypospadias repairs: a systematic review and pooled proportional meta-analysis of postoperative urethrocutaneous fistulas. J Pediatr Urol.

[14] Scarcia M., Maselli F.P., Cardo G., Ludovico G.M. (2016). The use of autologous platelet rich plasma gel in bulbar and penile buccal mucosa urethroplasty: preliminary report of our first series. Arch Ital Urol Androl.

[15] Kuo H.C. (2023). Intravesical injections of autologous platelet-rich plasma for the treatment of refractory interstitial cystitis. Low Urin Tract Symptoms.

[16] Suharyani S., Leonardo M., Oentoeng H.H. (2024). Efficacy and safety of platelet-rich plasma intracavernous injection for patients with erectile dysfunction: a systematic review, meta-analysis, and meta-regression. Asian J Urol.

[17] Iblher N., Fritsche H.M., Katzenwadel A. (2012). Refinements in reconstruction of penile skin loss using intra-operative prostaglandin injections, postoperative tadalafil application and negative pressure dressings. J Plast Reconstr Aesthetic Surg.

[18] Demzik A., Peterson C., Figler B.D. (2020). Skin grafting for penile skin loss. *Plastic and Aesthetic research***2020**.

